# Synergistic effects of sitagliptin and losartan against fipronil-induced hepatotoxicity in rats

**DOI:** 10.14202/vetworld.2021.1901-1907

**Published:** 2021-07-25

**Authors:** Sara T. Elazab, Omar Samir, Marwa E. Abass

**Affiliations:** 1 Department of Pharmacology, Faculty of Veterinary Medicine, Mansoura University, Mansoura,35516, Egypt; 2 Laboratory Animal Resource Center in Transborder Medical Research Center, Faculty of Medicine, University of Tsukuba, 1-1-1, Tennodai, Tsukuba, Ibaraki, 305-8575, Japan; 3 Department of Pathology, Faculty of Veterinary Medicine, Mansoura University, Mansoura, 35516, Egypt; 4 Department of Surgery, Anesthesiology and Radiology Faculty of Veterinary Medicine, Mansoura University, Mansoura, 35516, Egypt

**Keywords:** fipronil, hepatotoxicity, losartan, oxidative stress, sitagliptin

## Abstract

**Background and Aim:**

Fipronil (FPN) is a potent pesticide that is heavily used around the world in agriculture. However, its irrational use could potentially have deleterious effects on animals and humans. The present study aimed to investigate the ability of sitagliptin (Sit) and losartan (LOS), when used both individually or concurrently, to guard rat liver against the acute hepatotoxicity caused by FPN.

**Materials and Methods:**

Forty-two adult male Wistar rats were equally divided into seven groups (6/group). Group I (control) received normal saline (0.5 mL/rat, vehicle for all treatments) by gavage once daily for 10 days. Group II received oral Sit (10 mg/kg body weight [BW]) daily for 10 days and Group III received oral LOS (5 mg/kg BW) daily for 10 days. Group IV received oral FPN (19.4 mg/kg BW; 1/5 of the oral LD_50_) for the past 5 days of the study. Groups V and VI received oral Sit (10 mg/kg BW) and LOS (5 mg/kg BW) daily, respectively, 5 days prior and 5 days during FPN administration (19.4 mg/kg BW). Group VII received oral Sit (10 mg/kg BW) and LOS (5 mg/kg BW) for 10 days with daily FPN during the past 5 days. After the end of the treatment period, the rats were humanely sacrificed and blood and liver tissue samples were collected for biochemical analysis and histopathological and immunohistochemical investigations.

**Results:**

FPN administration resulted in elevated alanine aminotransferase, aspartate aminotransferase, and alkaline phosphatase serum concentrations as well as increased malondialdehyde levels and reduced catalase, superoxide dismutase, glutathione peroxidase, and glutathione activity. The histopathological investigation showed disorganization of the hepatic cords and focal necrosis of the hepatocytes in FPN-intoxicated rats. Furthermore, the immunohistochemical examination showed that hepatic caspase-3 was overexpressed in the FPN-treated rats. The administration of Sit and LOS before and alongside FPN markedly mitigated the alterations caused by FPN and the hepatoprotective effects were more prominent in the combination group.

**Conclusion:**

Sit and LOS, both individually or in combination, confers considerable hepatoprotection against FPN-induced hepatotoxicity.

## Introduction

Fipronil (FPN) is a strong and effective N-phenylpyrazole insecticide that is widely used in agricultural practices, as it is highly effective against several insects including flies, fleas, ticks, beetles, ants, and cockroaches [1]. FPN causes selective suppression of the insect nervous system and death, as it blocks the gamma-aminobutyric acid receptor and glutamate-activated chloride channels [2,3]. Despite its beneficial effects, the indiscriminate usage of FPN in large amounts can have harmful effects on mammals [4,5]. There is evidence that the toxic effect of FPN on humans is attributed to its metabolite, FPN sulfone, which affects the GABA-activated chloridechannels in mammals more than those in insects [6-9]. The previous reports have found that FPN has multiple toxic effects, such as thyroid impairment in rats [10], hepatotoxicity in humans and rats [11,12], and neurotoxicity in zebrafish embryos [13]. FPN reduces cellular antioxidants, causing oxidative damage in many tissues [12,14]. Hence, using exogenous antioxidants may mitigate FPN-induced toxicity in various organs.

Sitagliptin (Sit), a dipeptidyl peptidase-4 (DPP-4) inhibitor, is commonly prescribed for the treatment of adult-onset diabetes. DPP-4 is highly expressed in various organs and involved in the modulation of numerous body functions, such as chemokine and peptide production. Moreover, DPP-4 has also been found to play a major role in the procession of inflammatory changes and the determination of the immune response. Sit reportedly has antioxidant and anti-inflammatory effects [15-17]. Recently, Sit was also found to have hepatoprotective effects against xenobiotic toxicity [18,19]. Losartan (LOS) is an angiotensin II (Ang II) type I receptor (AT1R) blocker, widely used as a treatment for hypertension. LOS was found to abate oxidative injury in the liver by reducing the expression of Ang II-activated nicotinamide adenine dinucleotide phosphate hydrogen oxidase in the inflammatory areas of the hepatic tissue [20]. The ameliorative effect of LOS on the hepatotoxicity caused by carbon tetrachloride and paracetamol has been well documented [21,22]. Nevertheless, to the best of our knowledge, the effects of Sit and LOS on FPN-induced hepatic damage have not yet been investigated.

This investigation aimed to explore the potential ameliorative effects of Sit and LOS, both alone and concurrently, against FPN-induced hepatotoxicity in rats.

## Materials and Methods

### Ethical approval

The study protocol was approved by the Animal Ethics Committee of the Faculty of Veterinary Medicine, Mansoura University, Mansoura, Egypt (Approval No. R/21).

### Study period and location

The study was conducted in September 2019 at Faculty of Veterinary Medicine, Mansoura University, Mansoura, Egypt.

### Chemicals

FPN (FPN 20%; 5-amino-1-(2,6-dichloro-α, α, α-trifluoro-p-toyl)-4-(trifluoromethyl) sulfinylpyrazole-3-carbonitrile) was supplied from United Chemicals Company (Egypt). Sit (Januvia) was manufactured and supplied by Merck Sharp and Dohm Ltd. (Shotton Lane, Northumberland, NE 23 3 JU, Cramlington, UK). LOS was bought from Amriya Pharmaceutical Industries (Alexandria, Egypt). The kits used in the current study were purchased from Biodiagnostics Co. (Cairo, Egypt). All kits were used based on the manufacturers’ instructions in the enclosed pamphlets.

### Experimental animals

Forty-two adult male Wistar rats (100-125 g), obtained from the animal house in the Faculty of Pharmacy, Mansoura University, Egypt, were used in this study. They were supplied with rodentfood pellets and water *ad libitu*m and were kept at 25±2°C, under a 12 h light/dark cycle. Seven days were allowed for acclimatization before the experiment.

### Experimental design

The rats were equally divided into seven groups (six rats each). Group I (control) received normal saline (0.5 mL/rat, vehicle for all treatments) by gavage once daily for 10 days. Group II received oral Sit (10 mg/kg body weight [BW]) daily for 10 days [18] and Group III received oral LOS (5 mg/kg BW) daily for 10 days [23]. Group IV received oral FPN (19.4 mg/kg BW; 1/5 of the oral LD_50_ [24]) for the past 5 days of the study. Groups V and VI received oral Sit (10 mg/kg BW) and LOS (5 mg/kg BW) daily, respectively, 5 days prior and 5 days during FPN administration (19.4 mg/kg BW). Group VII received oral Sit (10 mg/kg BW) and LOS (5 mg/kg BW) for 10 days with daily FPN during the past 5 days.

### Sample collection

At the end of the treatment (11^th^ day), blood samples of 1.5 ml each were collected from the retro-orbital venous plexus of all animals and allowed to coagulate. The serum from each was separated and preserved at −20°C, until use in the liver function parameter analysis. Rats were then sacrificed by cervical dislocation. The liver was dissected and flushed with saline. A 0.5 g sample of the liver was homogenized from each specimen in 5 mL phosphate buffer saline (PBS; pH 7.4), centrifuged, and the supernatant was then kept at −80°C until use in the oxidative stress parameter evaluation for the liver tissues. Additional liver tissue samples were preserved in 10% formalin for the histopathological and immunohistochemical investigations.

### Biochemical analysis

The activity of aspartate aminotransferase (AST) and alanine aminotransferase (ALT) was assessed according to Reitman and Frankel [25], and serum alkaline phosphatase (ALP) activity was analyzed according to Tietz *et al*. [26]. Serum total protein was measured according to Bradford [27].

Oxidative stress biomarkers were screened, including malondialdehyde (MDA) as described by Uchiyama and Mihara [28], glutathione peroxidase (GPx) and reduced glutathione (GSH) as described by Paglia and Valentine [29], and catalase (CAT) and superoxide dismutase (SOD) as described by Nishikimi *et al*. [30] and Aebi [31].

### Liver histopathology and immunohistochemistry

Hepatic tissues were preserved in 10% formalin. They were then routinely processed until being embedded in paraffin blocks. Sections were cut to a 5 μm thickness andprocessed until being stained with hematoxylin and eosin according to Bancroft and Layton [32]. For immunohistochemical staining of the hepatic sections, the primary antibody against caspase-3 (polyclonal rabbit anti-cleaved caspase-3 at dilution 1:100, BioCare Medical, Cat: CP229C, Concord, CA, USA) was utilized. Immunostaining intensity was quantified using Image J software (National Institutes of Health, Bethesda, MD, USA) [33].

### Statistical analysis

The findings for all groups (n=6 rats/group) were shown as mean±SEM. All statistical analyses were conducted using SPSS, version 20 for Windows (SPSS Inc., Chicago, IL, USA). One-way analysis of variance, followed by Duncan’s multiple range *post hoc* test, was used to compare the data of various experimental groups. p<0.05 was considered statistically significant.

## Results

### Biochemical analysis

**Figure-1:**
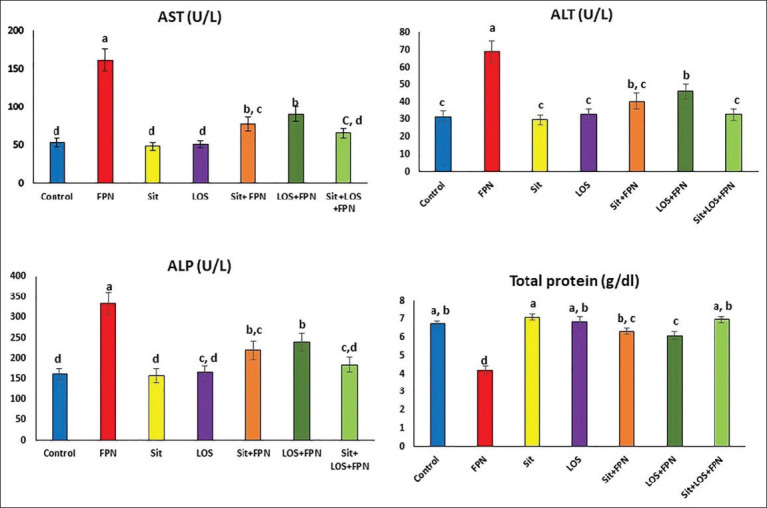
The effect of sitagliptin and losartan on serum concentrations of hepatic injury biomarkers in FPN-intoxicated rats. Data are presented as Mean±SEM (n=6). Means with different superscript letters (a-d) are statistically significant at (p≤0.05). FPN=Fipronil; Sit=Sitagliptin; LOS=Losartan; AST=Aspartate aminotransferase; ALT=Alanine aminotransferase; ALP=Alkaline phosphatase.

The biochemical parameters were measured to investigate alterations in hepatic function. Biochemical serum analysis showed statistically significant increases (p≤0.05) in the activity of AST, ALT, and ALP (204.3%, 119.1%, and 104.6%, respectively) and a significant reduction (p≤0.05) in total protein level (37.7%) in the FPN-treated group, when compared with the control. However, the Sit-FPN- and LOS-FPN-treated groups showed significant decreases (p≤0.05) in AST, ALT, and ALP when compared to the FPN-treated group (but still significantly higher than the control group). Total protein level was significantly elevated in the Sit-FPN- (50.7%) and LOS-FPN-treated rats (45%) when compared with the FPN-treated groups. While the Sit-LOS-FPN-treated rats exhibited restoration of the normal control levels of serum biomarkers (Figure-1).

**Figure-2:**
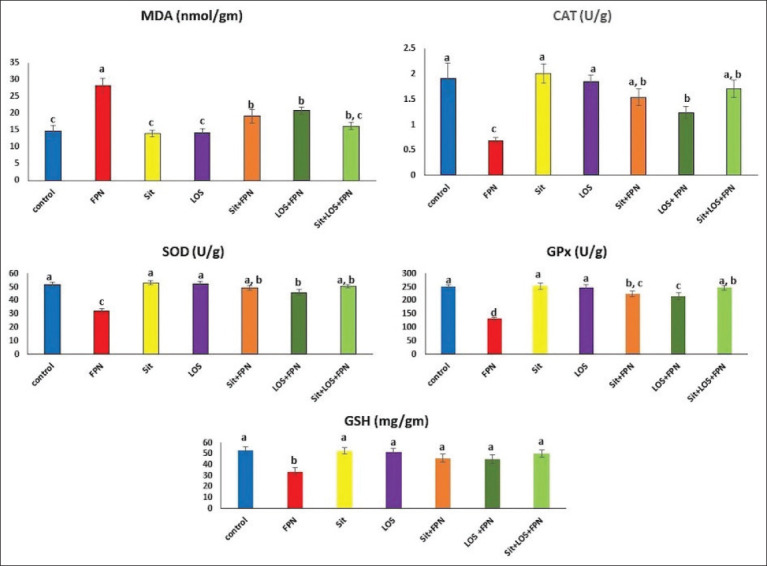
The effect of sitagliptin and losartan on hepatic tissue lipid peroxidation and activities of antioxidant enzymes in fipronil-intoxicated rats. Data are expressed as Mean±SEM (n=6). Means with different superscript letters (a-d) are significantly different at p≤0.05. FPN=Fipronil; Sit=Sitagliptin; LOS=Losartan; MDA=Malondialdehyde; CAT=Catalase; SOD=Superoxide dismutase; Gpx=Glutathione peroxidase; GSH=Reduced glutathione.

Biochemical tissue analyses showed that the administration of either Sit or LOS to the rats did not result in significant variations in MDA concentrations or CAT, SOD, GPx, and GSH activities relative to the control rats. On the other hand, the administration of FPN resulted in a significant increase (p≤0.05) in the level of MDA (91.1%) in the hepatic tissues as well as a significant (p≤0.05) reduction in hepatic CAT, SOD, GPx, and GSH activities (64%, 38%, 47.8%, and 37%, respectively). However, the Sit-FPN- and LOS-FPN-treated rats exhibited a significant (p≤0.05) decrease in MDA levels and an elevation in CAT, SOD, GPx, and GSH activities in the liver tissue when compared to the FPN-treated rats. Furthermore, the administration of the combined Sit and LOS treatment restored the normal levels of oxidant-antioxidant markers (Figure-2).

### Histopathological findings

**Figure-3:**
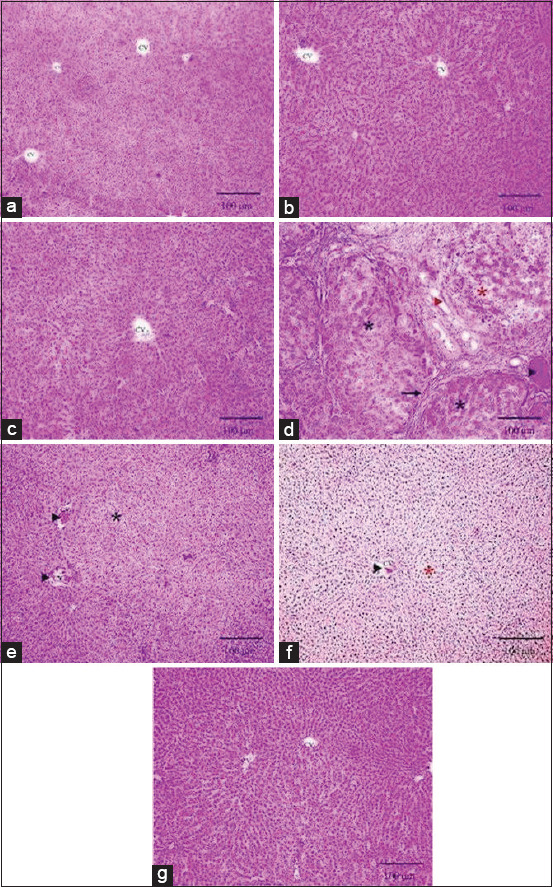
Light photomicrographs of hepatic tissues (sections stained with H and E). (a-c) Normally organized hepatic lobules consisted of radially arranged hepatic cords around central veins with normal sinusoids and portal areas. (d) Disorganization of hepatic cords (black asterisk) with the formation of broad fibrous septa separating the hepatic lobules and infiltrated with leukocytic cells (black arrow), besides congested blood vessels (black arrow head) and dilated lymphatics. In addition, the hepatocytes showing focal coagulative necrosis (red asterisk). (e) Mild congestion of the central veins (arrowhead) and mild diffuse hydropic degeneration of hepatocytes (black asterisk). (f) Mild congestion of central veins (arrowhead) and severe diffuse hydropic degeneration of hepatocytes (red asterisk). (g) Normal organization of hepatic cords and normal central veins. (a) Control, (b) sitagliptin (Sit), (c) losartan (LOS), (d) fipronil (FPN), (e) Sit-FPN, (f) LOS-FPN, (g) Sit-LOS-FPN scale bar =100 mm.

The photomicrographs from the control, Sit, and LOS group hepatic tissue sections showed normally organized hepatic lobules that consisted of radially arranged hepatic cords around central veins with normal sinusoids and portal areas (Figure-3a-c). In contrast, FPN-treated rats exhibited disorganization in their hepatic cords with the formation of broad fibrous septa separating the hepatic lobules and leukocytic cell infiltration, besides congested blood vessels and dilated lymphatics. In addition, the hepatocytes showed focal coagulative necrosis (Figure-3d). In the liver of the Sit-FPN-treated rats, mild congestion of the central veins and mild diffuse hydropic degeneration of the hepatocytes were noticed (Figure-3e). Similarly, LOS-FPN-treated rats showed mild congestion of the central veins and severe diffuse hydropic degeneration of the hepatocytes (Figure-3f). Interestingly, the hepatic tissue of Group VII (Sit-LOS-FPN) showed normal organization of the hepatic cords and normal central veins (Figure-3g).

## Immunohistochemical results

**Figure-4:**
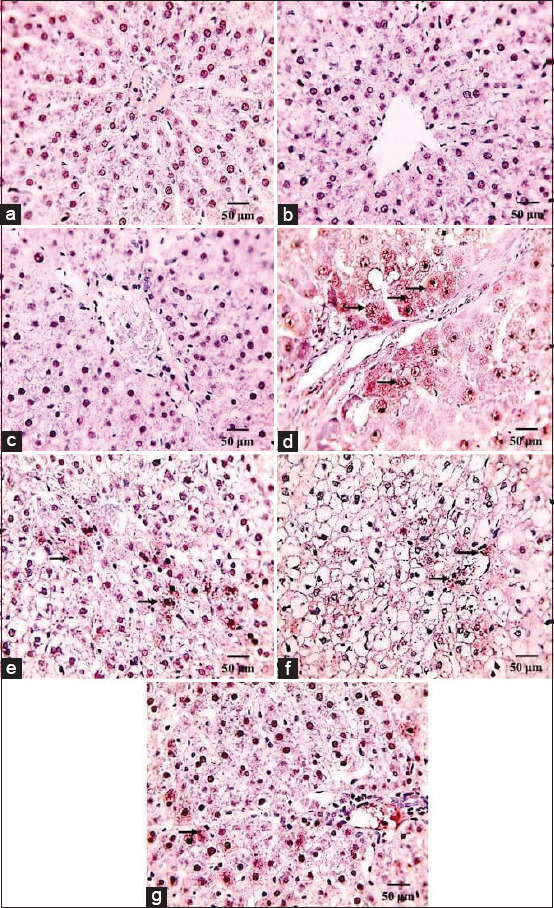
Immunohistochemical staining of rat liver by caspase-3. (a-c) Negative caspase-3 reaction in the liver sections. (d) Strong brown cytoplasmic signal of caspase-3 was observed in the hepatocytes from fipronil (FPN)-intoxicated rats (black arrow). (e) Moderate cytoplasmic signal of caspase-3 marker. (f) Moderate cytoplasmic signal of caspase-3 marker. (a) Control, (b) sitagliptin (Sit), (c) losartan (LOS), (d) FPN, (e) Sit-FPN, (f) LOS-FPN, (g) Sit-LOS-FPN scale bar =50 mm.

Immunohistochemistry showed a negative caspase-3 reaction in the liver sections of the control, Sit- and, LOS-treated rats (Figure-4a-c). On the other hand, a strong brown cytoplasmic signal for caspase-3 was observed in the hepatocytes of the FPN-intoxicated rats (Figure-4d). Meanwhile, moderate cytoplasmic signals of the caspase-3 marker were recorded in the hepatocytes of Sit-FPN- and LOS-FPN-treated rats (Figure-4e and f). Furthermore, Sit-LOS-FPN-treated rats exhibited mild cytoplasmic caspase-3 signals in the hepatocytes (Figure-4g).

## Discussion

The current study assessed the hepatotoxicity of FPN and the guarding effect of Sit and LOS therapies, both alone and concurrently, against FPN intoxication. The results showed that FPN treatment led to an increase in AST, ALT, and ALP activity in the serum of treated rats. The elevation of these enzymes may be associated with oxidative stress-mediated changes in the permeability of the hepatocytes, resulting in their leakage into the circulation system. The activities of these enzymes are serological hallmarks for hepatic toxicity [34]. These results agree with those from the previous investigations [12,14,24,35,36]. The deleterious effect on serum hepatic injury biomarkers was emphasized by the pathological lesions that were observed in the FPN-treated group. The histopathological investigation of hepatic tissues from FPN-treated rats showed that there was disorganization of the hepatic cords, the formation of broad fibrous septa separating the hepatic lobules, and leukocytic cell infiltration, besides congested blood vessels and dilated lymphatics, the hepatocytes also showed focal coagulative necrosis. In the same vein, a previous investigation recorded hepatocyte degeneration, congested central veins, and focal individual cell necrosis in the liver of rats exposed to FPN [24].

In accordance with the previous findings [24,37,38], we found an oxidant/antioxidant imbalance in the FPN-treated rats. FPN increased the MDA level, an indicator of lipid peroxidation that is associated with a significant depletion in the antioxidant enzyme levels (SOD, CAT, GPx, and GSH) in the livers of rats treated with FPN. These enzymes are crucial for cellular protection. For instance, SOD is responsible for quenching the superoxide radical to hydrogen peroxide (H_2_O_2_), which, in turn, is converted by CAT into H_2_O and O_2_, which are harmless products. These alterations in the antioxidant defense system of the hepatic tissue are probably due to the overproduction of free radicals by FPN and the exhaustion of antioxidant enzymes in its elimination, causing oxidative stress and consequent injury to the hepatocyte membrane [39,40].

Moreover, the immunohistochemical investigation of the liver revealed strong expression of the apoptosis marker, caspase-3, in the FPN group when compared to the control. This is in accordance with the previous investigations that reported that FPN increased caspase-3 in the hepatocytes isolated from rats [38] and that other pesticides, such as chlorpyrifos, caused apoptosis in human cell lines by stimulating caspase-3 [41,42]. The overexpression of caspase-3 may be linked to the increased hepatic oxidative stress and inflammation, indicating that FPN triggers oxidative stress and inflammation, which subsequently stimulates downstream apoptotic pathways.

Interestingly, the results of this study have elucidated the ameliorating role of Sit and LOS, both alone and concurrently, against FPN-induced hepatotoxicity in rats, as indicated by the restoration of normal control concentrations of the serum hepatic biomarkers and antioxidant enzymes in the rats treated with Sit and/or LOS. The protective effect of Sit may be attributed to its antioxidant and anti-inflammatory effects as well as its role in the inhibition of apoptosis through the modification of apoptotic and anti-apoptotic proteins [43-45]. These observations are in accordance with the previous research that found that Sit had a protective effect against hepatotoxicity induced by carbon tetrachloride and methotrexate [18,19]. Moreover, Sit has been shown to exert its hepatoprotective effect by potentiating the antioxidant status of the liver with the stimulating nuclear factor erythroid 2-related factor 2 (Nrf2) and suppressing nuclear factor kappa-B (NF-kB) [18], while, LOS may alleviate FPN-induced oxidative stress by acting as a quencher for oxygen free radicals [46]. Similarly, several reports have demonstrated the abrogative effect of LOS against the hepatotoxicity caused by carbon tetrachloride, thioacetamide, and paracetamol [21-23]. Our histopathological and immunohistochemical results also indicate that Sit and LOS have a protective effect against the hepatotoxicity induced by FPN.

## Conclusion

Sit and LOS, both individually and concurrently, confer noticeable hepatoprotection effects against FPN-induced toxicity. Future studies are warranted to gain a deeper insight into the mechanisms beyond the guarding effect of both Sit and LOS against FPN-induced hepatotoxicity and to investigate the safety and pharmacokinetic interactions between Sit and LOS.

## Authors’ Contributions

STE: Designed the study and wrote the manuscript. STE and MEA: Conducted the animal experiment and analyzed the data. OS: Performed histopathological and immunohistochemical investigations. All authors have read and approved the final manuscript.
